# Insect meal as a feed ingredient for poultry

**DOI:** 10.5713/ab.21.0435

**Published:** 2022-02-01

**Authors:** Usman Elahi, Chang-chun Xu, Jing Wang, Jing Lin, Shu-geng Wu, Hai-jun Zhang, Guang-hai Qi

**Affiliations:** 1 Key Laboratory of Feed Biotechnology, Ministry of Agriculture and Rural Affairs, National Engineering Research Center of Biological Feed, Feed Research Institute, Chinese Academy of Agricultural Sciences, Beijing 100081, China; 2 Institute of Animal and Dairy Sciences, Faculty of Animal Husbandry, University of Agriculture, Faisalabad 38000, Pakistan

**Keywords:** Broiler, Growth Performance, Immune Function, Insect Meal, Laying Hen, Meat Quality

## Abstract

Shortage of protein feed resources is the major challenge to the world farm animal industry. Insects are known as an alternative protein source for poultry. A wide range of insects are available for use in poultry diets. Insect larvae thrive in manure, and organic waste, and produce antimicrobial peptides to protect themselves from microbial infections, and additionally these peptides might also be functional in poultry feed. The feed containing antimicrobial peptides can improve the growth performance, nutrient digestibility, intestinal health, and immune function in poultry. Insect meal contains a higher amount of essential amino acids compared to conventional feedstuffs. Black soldier fly, mealworm, housefly, cricket/Grasshopper/Locust (*Orthoptera*), silkworm, and earthworm are the commonly used insect meals in broiler and laying hen diets. This paper summarizes the nutrient profiles of the insect meals and reviews their efficacy when included in poultry diets. Due to the differences in insect meal products, and breeds of poultry, inconsistent results were noticed among studies. The main challenge for proper utilization, and the promising prospect of insect meal in poultry diet are also addressed in the paper. To fully exploit insect meal as an alternative protein resource, and exert their functional effects, modes of action need to be understood. With the emergence of more accurate and reliable studies, insect meals will undoubtedly play more important role in poultry feed industry.

## INTRODUCTION

Fish meal and soybean meal are the conventional protein sources in poultry feed. In poultry production, feed cost is approximately 60% to 80% of the total cost. A possible solution to reduce poultry feed costs is finding available, efficient, and inexpensive alternative feed sources. Insects are natural foods for poultry. Chickens can be found picking worms, and larvae from the grass, soil, and litter where they walk.

Insects are capable of consuming animal manure, and food wastes, and reducing pollution, and providing protein (larvae), and fertilizer (frass). Insects convert waste into proteins, and reduce total nitrogen excretion, odors, and methane emission, thus reduce up to 80% of waste mass [1–3].

The use of insects in poultry feed is a potential solution to improve the sustainability of poultry diets. A wide range of insects are available for use in poultry diets [4]. Insect meal contains a greater amount of essential amino acids compared to conventional feedstuffs [4]. The insects can be used as a live (fresh), dried, and paste form for poultry diets [5,6]. A dried insect is considered suitable for poultry diet because the water content in fresh or live insect stimulates the degradation, antimicrobial activity, and Millard reaction [6,7].

The exoskeleton of insects mainly consists of chitin, which improves the immune system of chicken [4]; however, chicken cannot synthesize the chitin [8]. Chitin and chitin derivatives can stimulate the innate immune cells [9]. Chitin contains about 5% nitrogen [10,11]. Broiler chickens fed the diet containing mealworm meal (MWM) have better disease resistance, and immune response due to prebiotic effect of chitin [12]. Chitin in diet also halts the growth of *Escherichia coli*, *Salmonella*, and *Salmonella enterica* serovar Typhimurium in broiler chickens [13,14]. Furthermore, hypolipidaemic and hypocholesterolaemic properties of chitin produce leaner meat by decreasing body fat in broiler chickens [8]. However, insects are recognized as disease carriers and there is a threat that insect borne diseases could transfer to poultry, and humans [15]. Black soldier fly (BSF; *Hermetia illucens*) does not carry any disease-causing agent; however, housefly (HF; *Musca domestica*) is a carrier of *Entomophthora* spp. fungus, house cricket (*Acheta domesticus*) is a carrier of *Metarhizium* sp. fungus and cricket paralysis virus, and mealworm (*Tenebrio molitor*) is a carrier of *Beauveria bassiana* fungus [16]. Insect larvae produce antimicrobial peptides to protect themselves from microbial infections as well as these peptides could also be functional in poultry [3,17]. Moreover, proper processing of insects could reduce the chemical risks and makes it gluten free [18,19]. Insects also contain antimicrobial peptides that are active against microbial resistant, bacteria, viruses, fungi and parasites as well as being used in medicines for wounds, infections, cancer, flatulence, phlegm, spasms and anticoagulation [17]. Antimicrobial peptide P5 is antibiotic alternative which acts as a growth promoter [20]. In addition, antimicrobial peptides improve the growth performance, nutrient digestibility, gut health and immune functioning [21]. Furthermore, the dark color of insect cuticle is due to the bioactive phenolic compound melanin having antibacterial and antifungal activity as well as prevents and treats hepatic diseases, stress and tumors [22–24]. In addition, insects are enriched in fatty acids that have antimicrobial properties. One of which, Lauric acid, is known for antibacterial and antiviral activity [25].

Insect meal in poultry diets increases the palatability for chickens and chickens fed on insect meal are highly preferable by consumers [4]. Insect meal enhances immune system and reduces antibiotic use thus, promoting animal health [8]. Moreover, feeding grasshoppers to chickens improved the shelf life of the meat [26]. Using insect meal in diet reduces feed cost, and enhances the performance and health of broiler chickens [27]. Thus, insect meal is an acceptable, inexpensive, and preferable source of protein for poultry.

## BLACK SOLDIER FLY (*Hermetia illucens*) MEAL

Black soldier fly meal (BSFM) is a good source of protein, and energy, enriched with essential, and nonessential amino acids, saturated, monounsaturated, and polyunsaturated fatty acids (PUFA), vitamins, and minerals [10,28,29]. The concentration of crude protein (CP) in BSFM ranged from 35% to 61%. Reported values for CP are, 34.97% [10], 36.94% [30], 36.9% [31], 40% [32], 42.6% CP [33], 43.9% [34], 55.3% [11], 55.3% [35], 56.1% [36], and 60.8% [37–40]. Black soldier fly contains higher concentrations of lauric acid and palmitic acid [41]. The concentration of crude fat in BSFM ranged from 7% to 42%. Reported values for crude fat are, 6.84% [36], 14.1% [37–40], 18% [11,35], 29.4% [34], 32.5% [32], 34.3% [31], 35.49% [10], 36.9% [33], and 42.27% [30]. Methionine content in BSFM ranged from 0.08% to 0.90%. Reported values for methionine are, 0.08% [39], 0.50% [10], 0.60% [30], 0.64% [11], 0.75% [37,38,40], 0.80% [34], and 0.90% [31,36], however, methionine + cysteine is 1.30% [36]. The concentration of lysine ranged from 0.34% to 3.30%, and threonine ranged from 0.22% to 2.26%. Reported values for lysine are, 0.34% [39], 2.10% [10,11], 2.15% [30], 2.23% [31], 2.81% [34], 3.22% [36], and 3.29% [37, 38], 3.30% [37, 38,40], and, reported values of threonine are 0.22% [39], 1.52% [31], 1.63% [34], 1.72% [11], 2.17% [37,38,40], and 2.26% [36]. And reported concentration of valine in BSFM is 0.33% [39], 2.20% [31], 2.50% [34], 2.72% [11], 3.25% [37,38], 3.26% [37,38,40], and 3.38% [36]. Black soldier fly larvae contain 3 to 10 times higher calcium and magnesium content than other insects [42]. The concentrations of calcium and phosphorus in BSFM are ranged from 1.21% to 4.39%, and 0.74% to 0.95% respectively. Reported values for calcium are 1.21% [36], 2.46% [30], 4.39% [10], and for phosphorus are 0.74% [30], 0.83% [10], and 0.95% [36].

In addition, BSF larvae reduces manure mass by 50%, and total nitrogen concentration by 62% [43]. The rapid consumption of the substrate reduces odors and therefore presumably methane formation and off-gassing. Moreover, rearing BSF larvae on animal manure could help to reduce feed cost, HF population (by repelling ovi-position), pathogenicity (by producing certain enzymes) and odor [3,44,45].

Considerable studies showed that BSFM is the superior insect protein to improve growth performance, carcass composition, and meat quality in broiler chickens. Diet containing 2.6% BSFM with extended amino acids supply in the diet of Ross 308 broiler chickens improved the growth performance and nitrogen balance in starter phase [37]. Cobb broiler chickens fed diet containing 5% BSFM had improved feed efficiency, and 7.5% BSFM increased the thigh weight and reduced meat pH, and 10% BSFM still resulted in better growth [46]. Diet containing 20% BSFM fed to Ross 308 male broiler chickens improved the meat quality by increasing concentrations of lauric acid, myristic acid, and eicosapentaenoic fatty acid; however, partly reduced the total PUFA [32]. In another study, diet containing 5% BSFM fed to Ross 708 male broiler chickens improved the cecal microbiota population and preservation, and increased villi mucin; however, chickens fed on 15% BSFM had reduced cecal microbiota population and preservation [35]. Diet containing 5% BSFM fed to broiler chickens reduced the abdominal fat percentage; 10% BSFM increased the carcass weight and breast percentage, and 15% BSFM increased the body weight, abdominal fat percentage, meat redness, meat protein percentage, breast meat monounsaturated fatty acids (MUFA), and reduced breast meat PUFA [47]. Diet containing 5% or 10% BSFM fed to Ross 308 broiler chickens led to improved growth performance; however, 15% BSFM in diet decreased the feed efficiency, and resulted in the increase of crypt depth and reduction of villus height, and villus height crypt depth ratio [48]. Cobb 500 broiler chickens fed on the diet containing 2% BSFM had decreased abdominal fat weight; diet containing 6% BSFM increased the protein digestibility and reduced the excreta *Enterobacteriaceae* count; 8% BSFM had better growth performance, and 10% BSFM increased drip loss, and decreased the gizzard weight [49]. Indigenous Ardennaise chickens fed on 8% fresh BSF exhibited higher body weight [41]. Diet containing (3% or 6%) BSFM fed to Ross 308 broiler chickens increased the breast meat yield and feed efficiency; however, reduced the weight gain [20]. BSFM at the dosage of 3% exhibited immunomodulatory effects, as evidenced by the increase of the CD3+CD4+ T lymphocytes, cell proliferation, lysozyme, survivability against *Salmonella Gallinarum*, and decrease of the bacteria count in the tissues of liver, spleen, bursa, and cecum [50].

The effects of BSFM on broiler chicken were affected by the dietary ratio between methionine and cysteine. Ross 308 male broiler chickens fed on the diet containing 23%/21% BSFM (starter/grower phase) with 50:50 methionine cysteine ratio improved feed efficiency, increased net protein utilization and body crude fat deposition; diet with 40:60 methionine cysteine ratio reduced the growth performance, methionine precaecal digestibility, and net protein utilization; diet containing 60:40 methionine cysteine ratio increased amino acids (methionine, threonine, arginine, leucine and valine) precaecal digestibility; however, diet with 55:45 methionine cysteine ratio only increased the cysteine precaecal digestibility [39].

Black soldier fly meal can be used to replace fish meal and/or soybean meal or even soybean oil in broiler diet. Fish meal was successfully replaced by BSFM up to 15% in the diet of domestic chickens [51]. Cobb 500 broiler chickens fed on the diet containing 33% BSFM (4% in diet) as a replacement of fish meal resulted in increased dressing percentage and protein deposition in meat [52]. Ross 308 broiler chickens fed on the diet containing 50% or 100% BSFM with an ideal amino acid ratio as a replacement of soybean meal improved the growth performance, while diet containing 100% BSFM with deficient methionine level reduced the feed intake, protein and energy conversion ratio [40]. Cobb 500 broiler chickens fed diet containing 5% BSFM as replacement of soybean meal and fish meal, had better growth performance, and higher gizzard weight; and 10% BSFM increased the breast weight, and overall acceptability of cooked pectoral muscle; while 15% BSFM reduced the aroma, and taste of cooked pectoral muscle, total feed cost, and increased gross profit margin [34]. Ross 308 broiler chickens fed on the diet containing 75%/50% BSFM (75% for starter phase and 50% for grower phase), or 50% or 100% BSFM with extended amino acids supply as replacement of soybean meal improved growth performance and CP deposition; further, 50% BSFM and 100% BSFM with extended amino acids supply, yielded superior protein quality model parameter and net protein utilization [38]. BSFM was successfully included at 15% in the diet of Cobb 500 broiler chickens [53] and soybean oil was successfully replaced with 100% BSF fat for Ross 308 male broiler chickens [54]. Diet containing 100% BSFM as a replacement of soybean oil exhibited increased proportion of saturated fatty acids and reduced proportion of PUFAs of breast meat, and did not affect the growth performance, hematological parameters, carcass, and meat quality [55]. Black soldier fly fat could be used to replace 50% of soybean oil and reduced cholesterol in the breast meat in Ross 708 broiler chickens; while 100% BSF fat increased the total saturated fatty acids and reduced the MUFA, PUFA in breast and leg meat [56]. Ross 308 male broiler chickens fed diet containing 2.5% partially defatted BSFM resulted in increased digestibility of crude fat, and apparent metabolizable energy compared to chickens fed 2.5% full defatted BSFM [11]. BSFM included in the diet of Ross 708 broilers at 25% resulted in increased coefficient of total track apparent digestibility for ether extract compared to the chickens fed on 25% MWM diet [31]. BSFM could be used at 7.8% in combination with 5.2% alfalfa meal as a replacement of soybean cake to improve growth performance, carcass composition and meat redness in Hubbard S757 broilers [57].

There were few studies regarding the effects of BSFM in laying hens, and variable results were observed. Hy-Line Brown laying hens fed on 3% BSFM improved the growth performance, apparent digestibility of CP and crude fat, immunoglobulin A and glutathione peroxidase [10]. Laying hens (Julia) fed on the diet containing 10% BSFM increased the egg weight, albumin weight, egg shell thickness, albumin height, plasma calcium; furthermore, diet containing 10% BSF larvae meal significantly improved the egg yolk color score [28]. Lohman brown classic laying hens fed on diet containing 15% defatted BSFM and fat improved egg weight, egg mass, nitrogen, and energy metabolizability [58]. Xuefeng black-bone laying hens fed on 3% BSFM diet improved the egg weight, Haugh unit, egg shell weight, yolk C14:00, C17:00, C20:2 fatty acids, yolk amino acids (glutamic acid, methionine, phenylalanine and leucine), plasma total superoxide dismutase, and plasma avian influenza virus antibody, and decreased the egg shell thickness, and plasma interleukin-2; still, hens fed on 5% BSFM diet improved egg production, and feed efficiency, and decreased plasma malondialdehyde [30]. Diet containing 7.5% BSFM fed to Shaver white leghorn hens increased the feed intake, body weight (27 week), yolk color score, and shell thickness; however, 5% BSFM in diet increased body weight (23 week) and shank breaking strength but reduced the hen day egg production, egg weight, egg mass, and feed intake [36]. Lohman Brown Classic laying hens fed on the diet containing 17% BSFM to fully replace soybean meal resulted in poor growth, and production percentage, decreased blood lipids, blood chloride, and blood creatine; however, increased percentage of small, medium, and extra-large size eggs, blood globulin and blood calcium [59]. Diet containing 24% BSFM as replacement of soybean cake fed to Lohmann selected leghorn classic laying hens exhibited increases of the fecal dry matter [60].

## MEALWORM (*Tenebrio molitor*) MEAL

Mealworms are the brown worm-like larvae of the darkling beetles. Mealworms can be found throughout most of the world where they prefer warm, dark, and damp places like under decaying logs and leaves. Mealworms are designed for burrowing and eating and will feast upon the grains, vegetation, spoiled food, and many other types of fresh or decaying organic matter.

The concentration of CP in MWM ranged from 27% to 54%, and fat ranged from 4% to 34%. Reported values for CP concentration in MWM are 46.44% [61], 51.93% [12], 52.89% [6], 53.83% [62], 47% [63], 53% [64], 27.26% [65], 27.15% for super MWM [65], 45.83% [66], and 52.4% [31]. Reported values for crude fat in MWM are 21.27% [12], 30.05% [6], 28% [31], 28.03% [62], 29.6% [63], 3.6% [64], 11.50% [65], 8.70% for super MWM [65], and 34.2% [66]. Broiler chickens fed on the diet containing MWM have better disease resistance and immune responses due to prebiotic effect of chitin [12].

Arbor Acres broiler chickens fed on diet containing 2.5% MWM improved the weight gain (1 to 10 d) and reduced the albumen globulin ratio; however, 5% MWM reduced the albumen globulin ratio and intestinal *Escherichia coli* count [62]. Diet containing 4% MWM fed to Ross 308 male broiler chickens improved the body weight, average daily gain, and feed conversion ratio (FCR) in the starter phase [6]. Mealworm meal in diet of Ross 308 broiler chickens at the rate of 0.3% increased the weight gain, feed intake, blood total protein, blood total cholesterol, serum interleukin-2 and serum tumor necrosis factor α [63] and increased the cecal α-glucosidase [67]. Higher level of MWM (10% to 15%) in the diet of Ross 708 broiler chickens resulted in reduced firmicutes Bacteroidetes ratio and mucin synthesis [68]. Label Hubbard hybrid free range chickens fed on 7.5% MWM as a replacement of corn gluten meal in diet increased the oleic acid percentage and α-linolenic acid percentage, and reduced the atherogenicity and thrombogenicity indexes of breast meat [69]. Shaver brown male broiler chickens fed on diet containing MWM exhibited higher amount of volatile fatty acids of cecal content [70]. Ross 708 male broiler chickens fed on the diet containing 15% MWM as a replacement of soybean meal, corn gluten meal and soybean oil resulted in increased body weight (12 d), feed intake, FCR (25 to 53 d), and crypt depth, and reduced villus height, and villus height crypt depth ratio; however, body weight at 25 d was increased by 10% MWM and body weight at 53 d was increased by 5% MWM [71]. Diet containing 8% MWM fed to Ross 308 broiler chickens resulted in increased body weight, meat tenderness, and juiciness; however, decreased feed intake, and FCR [64]. Ross 708 female broiler chickens fed on diet containing 15% full fat MWM as a replacement of soybean meal, corn gluten meal and soybean oil exhibited increased body weight (12 d), and weight gain at 12 d, feed intake (1 to 12 d), thigh weight and abdominal fat weight; however, 5% MWM increased the body weight at 40 d, feed intake (12 to 25 d), and carcass weight [72]. In addition, 10% MWM increased the abdominal fat percentage and red blood cells; however, reduced the blood albumin and blood gamma glutamyl transferase [72]. Chickens fed a 3% MWM in diet exhibited increased weight gain, dressing percentage, feed cost, total expenses, gross return, and net profit [66]. Ross 308 male broiler chickens infected with *Salmonella* enteritidis and *Escherichia coli*, fed on the diet containing 0.4% MWM resulted in increased feed intake, serum IgA, and reduced mortality and cecal *Escherichia coli*; however, 0.4% super MWM increased the body weight, weight gain, serum immunoglobulin G, and reduced FCR, bursa of fabricius percentage, cecal *Salmonella* spp. [65].

Corn gluten meal in diets of female label Hubbard hybrid free-range chickens, was successfully replaced by 7.5% MWM without any effect [73]. Diet containing 29.65% MWM as a replacement of soybean meal with hulls in the diet of Shaver brown broiler chickens improved the FCR, ileal digestibility, and spleen weight [74]. Shaver brown male broiler chickens fed on diet containing 29.65% MWM showed improved FCR, protein efficiency ratio (PER), European efficiency factor, aspartate aminotransferase and alanine aminotransferase; however, reduced feed intake (46 to 62 d), albumin globulin ratio and uric acid [12]. Diet containing 25% MWM fed to the Ross 708 broiler chickens increased apparent ileal digestibility coefficient for isoleucine, lysine, methionine, phenylalanine, valine, alanine, aspartic acid, glycine, glutamic acid, and tyrosine compared to the chickens fed on the diet containing 25% BSFM diet [31]. Mealworm meal can also regulate the meat quality of poultry. The MWM sloth at 1% dosage reduced meat color redness, meat color yellowness, meat palmitic acid, palmitoleic acid, linoleic acid and saturated fatty acids, and increased meat oleic acid and unsaturated fatty acids [75]. Broiler chickens fed on the diet containing 1% MWM increased the body weight, weight gain; in addition, diet containing 2% MWM increased the carcass yield, slaughter weight, dressed weight, eviscerated weight, and reduced the abdominal fat weight; however, diet containing 10% MWM decreased the feed efficiency [76].

## HOUSEFLY (*Musca domestica*) MEAL

The HF can be found in all countries and in any climates. It is commonly associated with animal feces and can be found feeding on animal manure and food wastes. The concentration of CP in HF meal (HFM) ranged from 40% to 64%, and crude fat in HFM ranged from 2.5% to 28%. HFM contains about 53.3% CP [77], 54.36% CP and 16.90% crude fat [78], 55.1% CP and 20.7% crude fat [79], 55.6% CP and 27.9% crude fat [80], 59.48% CP and 6.66% crude fat [81], 63.99% CP and 24.31% crude fat [82], 61.25% CP [83], 44.44% CP and 9.76% crude fat [84], 48.4% CP and 20% crude fat [85], 62.98% CP and 5.58% crude fat [86], 40.12% CP and 6.88% crude fat [87], 50% CP and 2.7% crude fat [64]. The older HF larvae contain less CP and more lipids than young HF larvae [88,89]. The amino acid profile of HFM is comparable to fish meal, most limiting amino acids, lysine, and methionine are in higher concentration. Insect processing method could also influence the nutritional profile of the insect meal. Sun drying reduces CP and increases lipids compared to oven drying [89].

Housefly meal can be used as a substitute for fish meal or soybean meal, and HFM can improve the production performance [84,86,90–92] and meat quality of broilers [80, 93,94] at different concentrations. Diet containing 20% HFM as a replacement of fish meal fed to Anak broiler chickens increased the body weight, feed intake, FCR and gizzard percentage; however, diet containing 40% HFM increased weight gain, dressing percentage and inguinal fat percentage [84]. Ross 308 male broiler chickens fed 4% HFM diet had better growth performance, while dietary 8% HFM addition harmed the growth of starter [86]. Broiler chickens fed on the 60% HFM as replacement of soybean meal in diet, improved the body weight, FCR, dressing percentage, apparent metabolizable energy, nutrient digestibility and reduced the feed intake [90]. Yellow dwarf male chickens fed on 4.44% HFM as a replacement of fish meal in diet improved the weight gain and feed intake [91]. Diet containing 10% HFM fed to Ross 308 broiler chickens exhibited improved body weight (28 to 35 d), feed intake (28 to 35 d), weigh gain, FCR, European production efficiency factor (EPEF) and PER; however, diet containing 50% HFM reduced body weight (21 to 35 d), feed intake (21 to 35 d), weight gain, FCR, EPEF, and PER [92]. However, the HFM at the rate of 20%, 40%, and 60% was successfully replaced with fish meal in the diet of Ross 308 male broiler chickens without any significant effect [77]. Cobb 500 broiler chickens fed on the diet containing 5%q HFM for starter phase and, 4% HFM for grower and finisher phase, improved the body weight, weight gain, feed intake, FCR, meat flavor, meat aroma, meat desirability; moreover, 10% HFM increased the meat juiciness and flavor, and 20% HFM increased the meat tenderness and flavor [80]. Ross 308 broiler chickens fed on 10% HFM increased the body weight, carcass weight, breast muscle yield, juiciness, water holding capacity, and reduced the thawing loss and cooking loss [93]. Housefly live larvae was reported to have the potential to improve the reproductivity of free range chicken, as evidenced by the increased clutch size and hatchability [95]. Furthermore, 50% replacement of fish meal with HFM improved the hen day egg production in Isa brown and Nera black layer hens [96]. Table 1 summarizes the representative data of housefly meal application in broilers.

Everything has pros and cons. There are opinions that using maggot meal in poultry diets can enhance the risk of disease transmission. Houseflies are recognized as a carrier of diseases; they carry disease causing agents on their legs and hairs that cover their bodies. But the maggot itself doesn’t contain any disease-causing agent because maggot therapy has been used for decades for the treatment of septic injuries.

## CRICKET/GRASSHOPPER/LOCUST (*Orthoptera*) MEAL

Cricket/Grasshopper/Locust (Orthoptera) meal (OTM) is a rich source of protein, amino acids, fatty acids, minerals, and vitamins [97,98]. The concentration of CP ranged from 48% to 65% and crude fat ranged from 3% to 21%. Short horned grasshopper (*Oxya hyla hyla*) contains about 64.67% CP, 2.58% crude fat [97], reared African grasshopper (*Acanthacris ruficornis*) contains about 50.5% CP and 18.8% crude fat, desert locust (*Schistocerca gregaria*) contains about 50.9% CP and 20.5% crude fat, and wild edible grasshopper (*Ruspolia nitidual*) contains about 52% CP and 21.4% crude fat [99]. However, (*Ornithacris cavroisi*) grasshopper contains about 47.73% CP and 12.23% crude fat [100]. Chinese grasshopper (*Acrida cinerea*) contains about 65.4% CP and 8.3% crude fat [98]. Grasshopper contains about 52.50% CP and 27.1% crude fat [101].

Arbor Acres broiler chickens fed on the diet containing 50% (5% in diet) or 100% (10% in diet) grasshopper meal as fish meal replacer exhibited improved growth [100]. Grasshopper meal completely and successfully replaced fish meal in the diet of Anak 2000 broiler chickens without any effect [102] (Table 2). Qinjiaoma broiler chicken fed on the grasshoppers in pasture system resulted in increased heme iron, nonheme iron, total iron and α-tocopherol contents, and activities of glutathione peroxidase and superoxidase dismutase of breast and leg muscle compared to broiler chickens fed on control diet in cage system [103]. Isa Brown laying hens fed on diet containing 25% grasshopper (*Ornithacris cavroisi*) meal as a replacement for fish meal had improve Haugh unit, and diet containing 75% grasshopper (*Ornithacris cavroisi*) meal improved the egg yolk color [100]. Live grasshoppers fed to free range Qinjiaoma broiler chickens improved live weight, carcass composition, and total lipid, phospholipids, and anti-oxidative potential of meat [26]. Indigenous chicken fed on diet containing 50% wild edible grasshopper (*Ruspolia nitidual*) meal as replacement for fish meal improved the FCR and EE apparent digestibility, and diet containing 100% wild edible grasshopper (*Ruspolia nitidual*) meal improved the CP apparent digestibility; However, diet containing higher levels (above than 25%) of wild edible grasshopper (*Ruspolia nitidual*) meal as a replacement of fish meal resulted in reduced feed intake in indigenous chickens [99].

However, chitin and chitosan in OTM are not easily absorbed and utilized. Cobb 500 male broiler chickens fed on the diet containing 0.05% cricket chitosan or 0.05% cricket chitin displayed a negatively affected intestinal morphology and a downregulated mRNA expression of some nutrient transporters (PepT1, EAAT3, SGLT1, and SGLT5) [104].

## SILKWORM MEAL

The silkworm is the larva or caterpillar of a moth. The larvae spins the silk to make a cocoon where it pupates to the adult moth. Silkworms eat mulberry leaves and were native to northern China. The culture of silkworms is called sericulture. Silkworm meal is a good source of protein, fatty acids, amino acids, minerals and vitamins [105–107]. Silkworm contains about 71.9% CP [107] 45.87% for spun silkworm pupae and 50.31% for reeling silkworm pupae [108]. Silkworm chitin which is a component of exoskeleton, contains approximately 25% CP, it does not contain amino acids and is not digestible [107]. The reported values for fat are 20.1% for silkworm pupae meal [107], 7.94% for spun silkworm pupae and 25.76% for reeling silkworm pupae [108], 54% CP and 2.5% crude fat [64].

Different content of silkworm meal (SWM) can be used in poultry feed to replace fish meal or soybean meal. Silkworm meal successfully substituted for fish meal or soybean meal in the diet of broiler chickens with no significant effect [107–109]. Soybean meal was successfully and completely replaced by SWM in the diet of white leg horn hens without any effect [110].

Sonali chickens fed on the diet containing 25% SWM as replacement of soybean meal increased the weight gain, feed intake, heart percentage, breast meat yield, and reduced breast meat protein percentage and ash percentage; 50% SWM increased the meat pH, and n-3 PUFAs, and reduced the n-6 PUFAs of breast meat [111]. In addition, diet containing 75% SWM as replacement of soybean meal fed to Ross 308 broiler chicken resulted in increased body weight, feed intake, gross return/bird and profit/kg meat, and reduced cost/kg meat; 100% SWM has the opposite effect, and 25% SWM in diet reduced the feed intake and increased the cost/kg meat; 50% SWM reduced profit/kg meat [112] Table 3 lists the typical results of silkworm meal application in broilers.

## EARTHWORM MEAL AND VERMI-HUMUS

Earthworm meal (EWM) is rich source of protein, energy, and amino acids [113–115]. The concentration of CP in EWM ranged from 41% to 66%, and crude fat ranged from 3.5% to 18%. Reported values for CP are 63.06% [115], 65.68% for (*Eisenia foetida*) [116,118], 7.27% for vermi-humus [118], 55.87% [113], 57.85% [117], and 41.42% [114], and reported values for crude fat are 18.5% [115], 16.39% [113], 9.2% [114], and 3.5% [117]. Fresh earthworm (EW; *Lumbricus rubellus*) contains 6.89% CP and 2.25% crude fat [114]. It is generally believed that the CP content in earthworms is between 50% and 70%, and the crude fat content is less than 20%, and its content is related to the freshness and dryness of the earthworms. In addition, EW products are often used in poultry feed in the form of EWM or a mixture of EWM and vermi-humus.

Feeding broilers with feed supplemented with 1% EWM and 1% vermi-humus has a negative impact on the growth performance of broilers, although the immune functions were improved [118]. But the feed supplemented with 3% EWM and 1% vermi-humus can improve the performance of broilers and increase relative weight of immune organs, intestinal length, and intestinal lactic acid bacteria count [116]. Hybro G female broiler chickens fed on fresh EW (*Lumbricus rubellus*) diet improved the quality of meat for thigh and breast; in addition, diet containing 100% EWM (8% for 1 to 21 d, 5% for 22 to 35 d) as a replacement for fish meal reduced the fat content of breast, and thigh meat, and exhibited the higher acceptability of drumsticks [114]. Ningdu yellow female broiler chickens fed on diet containing 5% EWM had improved growth performance and antioxidant capacity [117].

Diet containing 3% EWM (*Eudrilus eugeniae*) improved the body weight gain, and diet containing 5% EWM improved the FCR and increased meat pH; and diet containing 7% EWM improved aroma, juiciness, residues, and flavor of the meat in Cobb 500 broiler chickens [119]. Ross 308 broiler chickens fed on the diet containing 2%, 4%, or 6% EWM increased the breast meat yield, high density lipoprotein level and reduced the low-density lipoprotein level, and increased body weight and feed intake were observed in diet containing 2% or 4% EWM [120]. It was reported that soybean and fish meals could be replaced partially with EWM between 10% to 15% in the broiler diets [121] (Table 4).

## TERMITE MEAL

Termite (*Sclerotized macropterous*) meal contains about 42.33% CP after Sun drying and 47.34% CP after roasting, and about 41% crude fat [122].

Termites (*Macrotermes subhyalinus* and *Macrotermes bellicosus*) were successfully substituted in dry or fresh form in the diet of indigenous chickens without any effect [123]. Inclusion of termites (*Glyptotermes montanus*) extracted endo-β-D-1,4-glucanase, avicelase, β-D-1,4-mannanase, β-D-1,4-xylanase and β-D-1,4-glucosidase enzymes in poultry diet can improve digestion in poultry [124].

## BEE MEAL

Bee slum contains about 9.37% CP and 54.9% crude fat [125]. Bee products are mainly used in poultry feed in three forms: bee propolis, bee pollen and bee slum. The appropriate dosage of bee products can influence the performance of broiler chickens, and a lower dose of Bee products can have a good growth-promoting effect when applied in poultry feed.

Ross 308 broiler chickens fed on the diet containing 0.025% bee propolis and 2% bee pollen increased carcass yield and reduced drip loss, skin yellowness, breast meat yellowness: and 0.05% bee propolis increased carcass yield and reduced drip loss, skin yellowness, breast meat yellowness [126]. Dietary addition of 0.05% to 0.1% bee propolis or 2% bee pollen increased the duodenal villi height, duodenal villi base width, villus height crypt depth ratio and reduced the duodenal villi crypt depth in broiler [127]. Diet containing 0.04% ethanol extracted bee propolis fed to Ross 308 broiler chickens increased the concentrations of glutamic acid, glycine and tyrosine in breast muscle, and aspartic acid, serine, alanine, tyrosine, histidine, and threonine in thigh muscle, and reduced the concentration of methionine in breast muscle and proline in thigh muscle; however, 0.04% ethanol extracted bee pollen reduced proline concentration in breast muscle [128]. Diet containing (0.04% or 0.08%) bee pollen fed to Ross 308 broiler chickens did not affect the blood mineral profile [129].

Ross 308 broiler chickens fed on the diet containing 0.04% bee pollen resulted in increased body weight and carcass weight [130]. Diet containing 25% or 50% bee slum as a replacement of corn in the diet of Anak 2000 broiler chickens reduced the body weight and feed intake and increased the pancreas percentage [125]. Ross 308 boiler chickens at week 3 exhibited improved body weight and feed intake when fed on diet containing 2,000 ppm pine originated bee propolis; however, diet containing 4,000 ppm have the opposite effect. Thus. higher level of pine originated bee propolis had adverse effect on growth performance and protein digestibility [131].

Lohmann LSL laying hens fed on the diet containing 0.025% and 0.05% bee propolis increased the egg mass, egg production, Haugh unit, albumen height, yolk height, yolk index, yolk weight, blood total protein, blood globulin, hemoglobin, lymphocytes and reduced FCR, yolk diameter, blood cholesterol, heterophil, heterophil lymphocyte ratio [132]. Diet containing 0.05% and 0.15% bee pollen fed to Sinai laying hens increased the egg number, egg mass, production percentage, feed intake, red blood cells, white blood cells, lymphocytes and reduced the body weight, weight gain, heterophils, heterophil lymphocyte ratio, blood cholesterol, blood triglycerides; however, diets containing 0.1% bee pollen have the opposite effect [133].

## CHALLENGE AND PERSPECTIVES

Although considerable studies have been conducted in broilers and laying hens, there are still some obstacles to the proper and efficient utilization of insect meal in poultry industry. The quality and nutrient profile of insect products varied with the differences in insect species, rearing medium, environment, and processing method. The absence of large-scale production and stable supply of insect meal do not favor the accurate evaluation of metabolizable energy and effective nutrient availability. Furthermore, the processing method needs to be updated for cost reduction of insect products, and risk reduction of pathogenic contamination and disease spreading. With the availability of quality and stable insect meals, more reliable efficacy studies may be performed to evaluate the health, immunomodulatory and functional effects of insect meals compared with the alternative protein feedstuffs.

In the current environment with studies of insect meal in broiler and laying hens, BSFM, MWM, and HFM exhibit the most promising industrialization prospects. However, earthworm, silkworm, and locust swarms can be effectively utilized in poultry feed. Because insects are used as medium of medicines for centuries, it is reasonable to believe that insects can be used in poultry diet to replace antibiotics because of their antimicrobial properties. Insect meal can also be used in low CP diets for amino acids adjustment as insects are enriched in essential amino acids. With the emergence of more accurate and reliable studies, insect meal will inevitably play a greater role in the poultry feed industry.

## Figures and Tables

**Table 1:** Application of housefly meal with different addition levels in broilers

References	Inclusion level (%)	Replacement/Alternative of	Percentage in diet	Poultry type	Results
[82]	0 5 10 15 20	Soybean meal	32%	Ross male commercial broiler chickens	5% HFM improved dressing percentage (p≤0.05) 10% HFM improved breast muscle yield (p≤0.05) 15% HFM improved live weight (p≤0.05), FCR (p≤0.05) and thigh muscle yield (p≤0.05) Muscle amino acids concentrations (p≤0.05)
[96]	0 25 75 100	Fish meal	25%	Isa brown and Nera black layer hens	50% HFM improved the hen day egg production (p≤0.05) and reduced the shell thickness (p≤0.05) 100% HFM reduced the shell weight (p≤0.05)
[79]	0 25 50 75 100	Fish meal	4%	Anak 3,000 broiler chicken	50% HFM reduced the Weight gain (p≤0.05) and Nitrogen retention (p≤0.05) 75% HFM reduced the Protein efficiency ratio (p≤0.05)
[95]	Live larvae	-	-	Free range chickens	Weight gain (p≤0.05) Clutch size (p≤0.05) Hatchability (p≤0.05) Egg weight (p≤0.05) Chick weight (p≤0.05)
[83]	0 5 10 15 (Brooding phase) and 0 50 100 (Grower-finisher phase)	Fish meal	4.5% (Brooding phase) 2% (Grower-finisher phase)	Arbor Acers broiler chickens	15% HFM improved weight gain (p≤0.05), feed intake (p≤0.05), FCR (p≤0.05) and feed cost (p≤0.05) at brooding phase 50% HFM increased the feed intake (p≤0.05) and abdominal fat (p≤0.05) at grower-finisher phase 100% HFM improved the weight gain (p≤0.05), FCR (p≤0.05), feed cost (p≤0.05), carcass yield (p≤0.05), heart percentage (p≤0.05), liver percentage (p≤0.05), gizzard percentage (p≤0.05) and leg percentage (p≤0.05) at grower-finisher phase
[96]	-	Fish meal	-	Qingyuan chickens	HFM improved meat quality
[81]	0 50 (Dried) 100 (Dried and fresh)	Fish meal	5% (Starter phase) 3% (Grower phase)	Hybro-G broiler chickens	Fresh HF larvae improved the body weight (p≤0.05) and weight gain (p≤0.05) 100% dried HFM improved weight gain (p≤0.05) at 22 to 35 day

HFM, housefly meal; FCR, feed conversion ratio.

**Table 2:** Application of *Orthoptera* meal with different addition levels in broilers

Reference	Inclusion level (%)	Specie	Replacement/alternative of	Percentage in diet	Poultry type	Results
[102]	0 5 10 15	Chinese grasshopper (*Acrida cinerea*)	Fish meal	10%	Arbor acres broiler chickens	Successfully replaced with no significant effect

**Table 3:** Application of silkworm meal with different addition levels in broilers

References	Inclusion level (%)	Replacement/Alternative of	Percentage in diet	Poultry type	Results
[109]	0 25 50 75 100	Fish meal	6.87%	Anak broiler chickens	Successfully replaced with no significant effect
[110]	0 6 8	-	-	RIR layer hens	6% SWM improved the live weight (p≤0.05) and FCR (p≤0.05), egg production (p≤0.05) and reduced the feed intake (p≤0.05) and feed cost (p≤0.05) 8% SWM improved the survivability (p≤0.05)
[111]	0 33 66 100	Fish meal	6%	Arbor Acers broiler chickens	100% SWM improved live weight (p≤0.05), FCR (p≤0.05) and profitability (p≤0.05), and reduced feed intake (p≤0.05)

SWM, silkworm meal; RIR, Rhode Island red; FCR, feed conversion ratio.

**Table 4:** Application of earthworm meal with different addition levels in broilers

References	Inclusion level (%)	Specie	Replacement/alternative of	Percentage in diet	Poultry type	Results
[121]	0 5 10 15 20	*Lumbricus rubellus*, *Perionyx excavatus*	-	-	Ross male broiler chickens	10% EWM improved body weight (p≤0.05) 5% EWM improved feed intake (p≤0.05) 15% EWM improved FCR (p≤0.05) 20% EWM reduced the fecal lactic acid bacteria count (p≤0.05)

EWM, earthworm meal; FCR, feed conversion ratio.
